# Genetics of Inner Ear Malformations: A Review

**DOI:** 10.3390/audiolres11040047

**Published:** 2021-10-12

**Authors:** Davide Brotto, Flavia Sorrentino, Roberta Cenedese, Irene Avato, Roberto Bovo, Patrizia Trevisi, Renzo Manara

**Affiliations:** 1 Section of Otorhinolaryngology—Head and Neck Surgery, Department of Neurosciences, University of Padua, 35128 Padua, Italy; flavia.sorrentino020@gmail.com (F.S.); roberta.cenedese@gmail.com (R.C.); roberto.bovo@unipd.it (R.B.); patrizia.trevisi@unipd.it (P.T.); 2 Department of Diagnostic, Paediatric, Clinical and Surgical Science, University of Pavia, 35128 Pavia, Italy; avato.irene@gmail.com; 3 Neuroradiology Unit, Department of Neurosciences, University of Padua, 35128 Padua, Italy; renzo.manara@unipd.it

**Keywords:** inner ear malformations, hearing loss, genetics, complete labyrinthine aplasia, common cavity, cochlear aplasia, incomplete partition, cochlear hypoplasia, posterior labyrinth

## Abstract

Inner ear malformations are present in 20% of patients with sensorineural hearing loss. Although the first descriptions date to the 18th century, in recent years the knowledge about these conditions has experienced terrific improvement. Currently, most of these conditions have a rehabilitative option. Much less is known about the etiology of these anomalies. In particular, the evolution of genetics has provided new data about the possible relationship between inner ear malformations and genetic anomalies. In addition, in syndromic condition, the well-known presence of sensorineural hearing loss can now be attributed to the presence of an inner ear anomaly. In some cases, the presence of these abnormalities should be considered as a characteristic feature of the syndrome. The present paper aims to summarize the available knowledge about the possible relationships between inner ear malformations and genetic mutations.

## 1. Introduction

Inner ear malformations are estimated to be present in around 20% of patients with congenital sensorineural hearing loss [1].

The relationship between inner ear anomalies and hearing impairment has been known since the first anatomical report in 1791 by Carlo Mondini, of Italy, and many distinct malformations have been described in the last 230 years [2].

A milestone in the evolution of the understanding and treatment of these anomalies is the classification system proposed and recently revised by Sennaroglu and coauthors [1,3]. This classification is world famous and is summarized in Table 1.

The knowledge about inner ear malformations increases every year, both in terms of the understanding of their morphological characteristics and the development of rehabilitative options. What in the past were considered malformations without hope of rehabilitation are now considered a conditions that can be easily managed in experienced centers. For the most severe malformations (such as Michel deformity or cochlear aplasia), a brainstem implant is now considered an available option [4,5], while for most of the other malformations, rehabilitation can be performed with hearing aids until a feasible cochlear implantation is available [6]. In some cases (i.e., common cavity deformities), it is still debated which procedure can offer the best results in terms of auditory outcome, but multiple options are available [7].

The classification system proposed by Sennaroglu et al. has the merit of collecting the anomalies described over the previous two centuries, extending the system proposed by Jackler et al. [8], and connecting the morphological characteristics of the malformations to the possible moment of the developmental arrest in the embryo.

In the literature, multiple hypotheses have been proposed about the pathogenesis of these malformations with a possible genetic etiology among them.

In recent years, the increasing availability of genetic consultation and testing has provided new data about the possible connection between inner ear malformations and genetic anomalies. In addition, the evolution of neuroradiological imaging with new computed tomography (CT) methods and magnetic resonance imaging (MRI) provided new data about syndromic patients. Indeed, in some syndromes, the well-known presence of hearing loss has been associated with the presence of inner ear malformations, in some cases extremely specific ones.

The detection of a genetic mutation is important in the evaluation of the possible risk of recurrence of the clinical condition in future pregnancies and to suggest a neuroradiological study of the hearing system. Conversely, the identification of inner ear malformations may suggest the performance of genetic testing and a search for extra-auditory features of a possible syndrome.

The present paper aimed to summarize the current understanding about the genetics of inner ear malformations (see Table 2).

## 2. Complete Labyrinthine Aplasia

Complete labyrinthine aplasia is an inner ear malformation characterized by the total absence of cochleovestibular structures, accounting for approximately 1% of cases of cochlear malformations [8]. It is also called Michel aplasia because it was first described in 1863 by Eugéne Michel [2]. It is supposed to be determined by the arrest of development of the otic placode in the early third week of gestation [1].

This malformation is the cause of a profound sensorineural hearing loss that cannot be rehabilitated with cochlear implants: The only available option is a brainstem implantation [5].

Radiological findings vary from petrous aplasia to otic capsule hypoplasia and the malformation may also be associated with anomalies of other temporal bone and skull base structures [9].

Complete labyrinthine aplasia is more frequently bilateral [10,11] but unilateral cases are also reported in literature [12], for example in a case of Wildervanck syndrome [13].

The only syndromic genetic cause of complete labyrinthine aplasia is LAMM syndrome, an autosomal recessive disorder characterized by this bilateral inner ear malformation, microtia, and microdontia. LAMM is caused by mutations in the FGF3 gene [14,15]. The recessive FGF3 mutations lead to a certain variability ranging from fully penetrant LAMM syndrome to deafness with residual inner ear structures and, by extension, with minimal syndromic manifestations [16]. Several families have been described with this condition and several novel mutations have been reported [17] in more than 60 individuals from more than 20 unrelated families [18,19]. Bilateral complete labyrinthine aplasia was also recently identified in one patient with HOXA1 mutation syndrome in association with bilateral internal carotid artery aplasia, developmental delay, and gaze abnormalities [20].

## 3. Rudimentary Otocyst

A rudimentary otocyst can be described as a small otic capsule remnant with cystic appearance and no connection to the brainstem since no internal auditory canal is present; also, the internal carotid artery seems to be missing in these cases [21]. Michel described this condition, in addition to describing complete labyrinthine aplasia, and it was included in Sennaroglu’s classification of inner ear malformations [2]. Similarly, to complete labyrinthine aplasia, it is supposed to be caused by a developmental arrest, later in the third week of gestation, after the otic vesicle has developed. Sometimes rudimentary semicircular canals can be found [21].

No information is available in the literature about genetic mutations causing rudimentary otocysts.

## 4. Common Cavity

**Figure 1:**
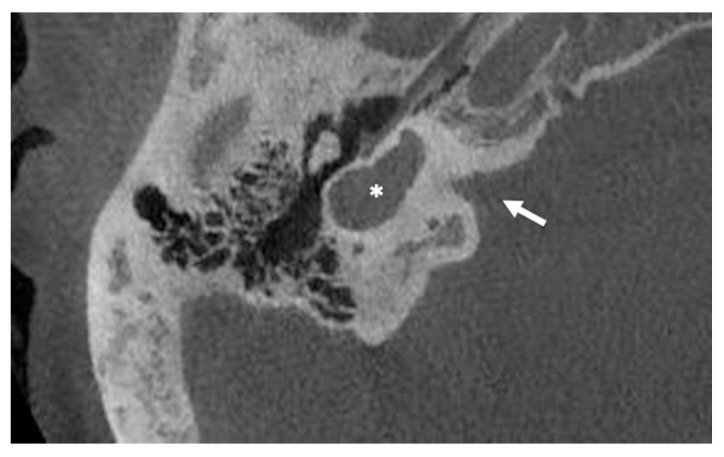
Cone beam CT imaging of a common cavity deformity (white asterisk). Note that the axis of the internal auditory canal (white arrow) seems to divide the cochleovestibular structure into anterior and posterior parts. This characteristic makes the common cavity different from the cochlea aplasia with dilated vestibule.

The common cavity is characterized by the absence of the normal differentiation between the cochlea and vestibule with the result of a single cystic cavity (see Figure 1).

Common cavity is supposed to be the result of a developmental arrest in the fourth week of gestation and accounts for about 0.7–26% of cochlear malformations [7].

Common cavities are assumed to have a non-genetic origin. However, two studies might suggest otherwise: one study found an association between common cavity deformity and autosomal recessive truncating HOXA1(P49639) mutations in an Bosley–Salih–Alorainy syndrome variant [22], another study described the case of a family with two children with congenital autosomal recessive non-syndromic sensorineural hearing loss and a common cavity anomaly in which an ROR1 gene mutation was identified [23]. A single case of common cavity was also described in a patient with 22q11 deletion syndrome [24].

## 5. Cochlear Aplasia (with and without a Dilated Vestibule)

**Figure 2:**
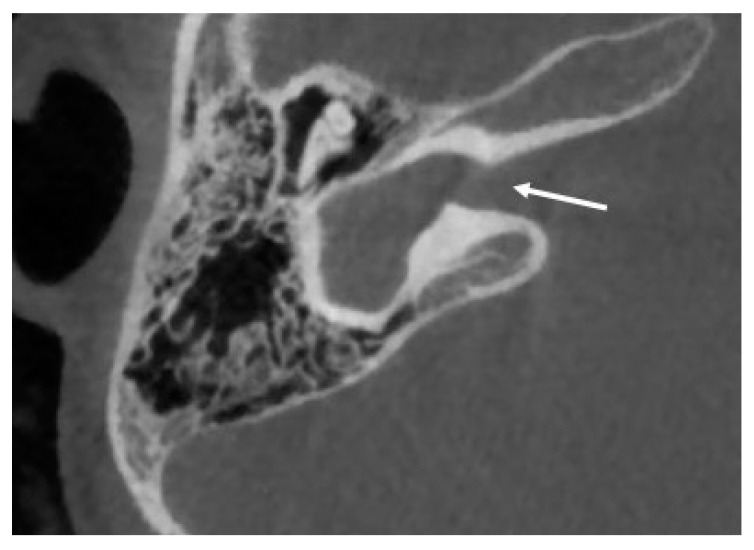
CT imaging of a cochlear aplasia with dilated vestibule. In contrast with a common cavity, the whole cochleovestibular structure is behind the axis of the internal auditory canal (white arrow).

Cochlear aplasia is characterized by a complete absence of the cochlea, with absence of the cochlear nerve canal and cochlear nerve, and it is supposed to account for only 3% of cochlear malformations (see Figure 2) [25].

Two subtypes can be identified of this anomaly: with and without dilated vestibule [1]. This malformation causes a profound sensorineural hearing loss. The possibility of cochlear implantation is controversial.

No specific gene mutations responsible for cochlear aplasia are reported in the literature, but PAX genes were supposedly involved in the development of the inner ear from experimental studies on animal models, in particular anomalies in gene PAX2 [26].

## 6. Incomplete Partitions

**Figure 3:**
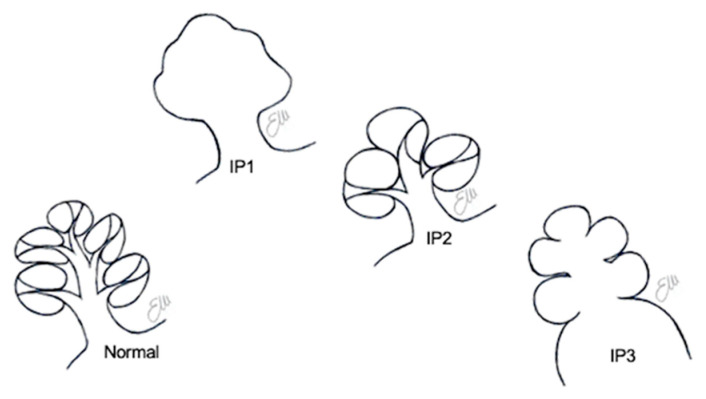
Schematic drawings of the three different types of incomplete partitions.

The incomplete partitions are three different anomalies in which there is a clear differentiation between the cochlea and the vestibule, the dimensions of the cochlea are normal, but the internal architecture of the cochlea is altered in different forms (see Figure 3) [1]. They are estimated to represent 40% of cochlear malformations [1].

Before 2002, these malformations were considered a single entity and named Mondini [2]; the current subdivision is due to Sennaroglu and Saatci who described the incomplete partition types 1 and 2 [3]. The incomplete partition type 3 was first described in 1971 as X-linked deafness [27] and only lately was it relabeled with the current name [3].

### 6.1. Incomplete Partition Type 1

**Figure 4:**
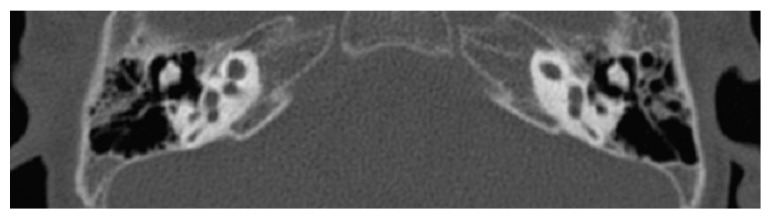
CT imaging of a bilateral incomplete partition type 1. The anterior (cochlear) and posterior (vestibular) parts of the inner ear are clearly distinguishable, with the cochlear portion being cystic and almost normal in terms of size.

The incomplete partition type 1 can be described as a cochlea with normal dimensions, divided from the vestibule and the posterior labyrinth (which can be altered or normal) with an extremely altered internal architecture. Indeed, the cochlea presents a cystic morphology that seems to be empty, because modiolus, interscalar septa, and lamina spiralis are almost missing (see Figure 4) [1].

This malformation causes a profound sensorineural hearing loss that requires cochlear implantation, and its prevalence is about 2% of all cochlear malformations.

No specific genes are reported in the literature that appear to relate to the etiology of the incomplete partition type 1. The current opinion is that an altered vascular supply should be considered the plausible cause of this malformation [1].

### 6.2. Incomplete Partition Type 2

Incomplete partition type 2 was identified as a distinct entity from incomplete partition type 1 in 2002 [3], but it is the first inner ear malformation ever described [2].

**Figure 5:**
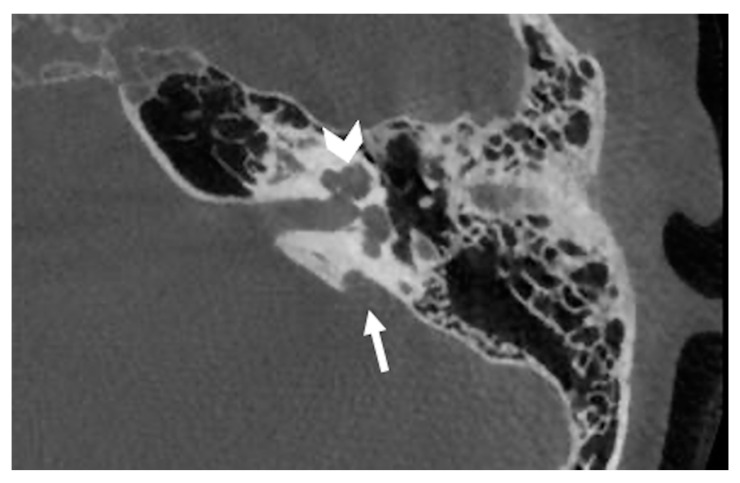
CT imaging of a left incomplete partition type 2. In this malformation the middle and apical turns appear to be fused (white arrowhead). Frequently, an enlarged vestibular aqueduct is also detectable (white arrow).

In this condition, the cochlea has a normal size, the basal turn is normal, and the upper part of the cochlea is altered. Indeed, the middle and apical turns form a cystic apex appearing to be fused and the apical part of the modiolus is missing, with variable anomalies of the upper interscalar septum. In addition, in half of the patients the posterior labyrinth may also present anomalies, specifically a dilated vestibule and anomalies of the semicircular canals (see Figure 5) [3].

What was described by Mondini in 1791 was a combination of this cochlear anomaly with the dilation of the vestibule and an enlarged vestibular aqueduct. Although the eponym Modini was used for decades as a synonym of “inner ear malformation”, clinicians and researchers should be aware that today the correct term for the cochlear malformation is incomplete partition type 2, and Mondini should be used to describe the combination of the three abnormalities. It is important to note that while reading the related literature, it is often not easy to understand if the authors use the correct terminology, so even in recent studies, the term Mondini should be considered with caution.

The enlargement of the vestibular aqueduct causes a mixed hearing loss, that is estimated to be stable in around 60% of the patients (with a third of them presenting fluctuations during life); the hearing deteriorates over time in around 40% of patients, in some cases with concomitant vestibular symptoms. A bilateral enlarged vestibular aqueduct is six times more common than a unilateral one [28]. It is difficult to estimate these data for isolated incomplete partition type 2.

Incomplete partition type 2 is detectable by imaging in patients with Pendred syndrome in which it is present as part of the Mondini triad along with thyroid goiters secondary to iodine organification defects [29]. Biallelic mutations in SLC26A4 lead to Pendred syndrome [30,31].

Enlarged vestibular aqueducts, with or without incomplete partition type 2, are frequently observed in patients with alterations of the SLC26A4/pendrin gene that is located on chromosome 7q31 and expressed in ears, thyroids, and kidneys [32].

According to the recent literature, the prevalence of SLC26A4 mutations in patients with enlarged vestibular aqueducts is variable among different ethnic groups. Biallelic mutations in SLC26A4 were found to range from the 57% to the 95% in Asian groups [33,34,35,36,37,38,39,40]. In Caucasian cohorts it is estimated that only 25% of patients with enlarged vestibular aqueducts present biallelic mutations [41], while another 25% present with monoallelic mutations [42,43], thus leaving one half of the patients without known genetic etiology of their anomaly.

The isolated enlarged vestibular aqueduct was described to also be related with mutations of FOXI1 (a gene in strict relationship with SLC26A4) and KCNJ10 [44].

### 6.3. Incomplete Partition Type 3

This incomplete partition is also known as X-linked deafness as it was first described by Nance et al. [27]. It is supposed to be the least frequent among the incomplete partitions, accounting for 2% of them [45]. This anomaly causes a mixed hearing loss, frequently worsening over time, with initial benefit from a hearing aid, but eventually requires cochlear implantation [46].

**Figure 6:**
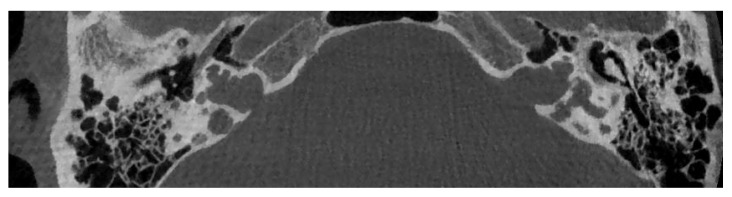
CT imaging of a bilateral incomplete partition type 3. The cochlear structures appear to be symmetrically altered, with the typical appearance of an empty cochlea, without a clear fundus of the internal auditory canal.

The incomplete partition type 3 is easily recognizable with CT and MRI, being characterized by a cochlea with normal external dimensions, but the modiolus is completely absent as is the lamina cribrosa at the fundus of the internal auditory canal. In addition, the internal auditory canal seems to be dilated and the stapes footplate is fixed (see Figure 6) [47].

This malformation was likely the first to relate to a specific inheritance. Indeed, since the first report it was identified as X-linked and now it is known to be caused by a POU3F4 gene mutation [48]. This gene is involved in neural tube development including what becomes the anterior region of the hypothalamus [32,49]. This explains why hypothalamic dysmorphisms were identified in patients with incomplete partition type 3 [50].

## 7. Cochlear Hypoplasia

The term cochlear hypoplasia accounts for a heterogeneous group of malformations characterized by a small external size, when compared to the average measures of the population, and a lower-than-normal number of 2½–2¾ turns [51]. These anomalies are estimated to be between 15% [25] and 23% of all inner ear malformations [52]. They present different audiological features and multiple rehabilitative options are available [52].

The current classification system [1,47] accounts for four subtypes of cochlear hypoplasia, previously described in numerous studies: (1) cochlear hypoplasia type 1: small budlike cochlea without an internal architecture; (2) cochlear hypoplasia type 2: a cochlea with an architecture similar to normal, reduced in size, with a modiolus or interscalar septa being present but defective; (3) cochlear hypoplasia type 3: internal and external architecture quite normal, but the size is reduced with fewer or shorter turns; and (4) cochlear hypoplasia type 4: a cochlea reduced in size with a normal basal turn and hypoplasia of the middle and apical turns [51,53].

Even if this classification describes four different subtypes of cochlea hypoplasia, most articles available in the literature categorize these anomalies with the general term of “cochlear hypoplasia” without further characterization. The above-mentioned limit explains why the connection between cochlear hypoplasia and genetic anomalies is difficult, and the relation between cochlear hypoplasia subtypes and genetic anomalies is currently impossible to describe.

Cochlear hypoplasia has been identified in branchio-oto-renal syndrome as a frequent feature in these patients [54,55] and can be detected in patients with oculo-auriculo-vertebral spectrum disorder [56].

Recently, a de novo missense variant [p(Asn174Tyr)] in the DNA-binding homeodomain of SIX1 was identified in a single patient with bilateral hearing loss due to cochlear hypoplasia and cochleovestibular nerve abnormality. This gene was previously associated with autosomal dominant hearing loss and branchio-oto-renal or branchio-otic syndrome [57].

Cochlear hypoplasia type 3 was identified in Waardenburg syndrome and was found to occur more frequently in probands with SOX10 variants [58]. According to some authors, 8% of patients with Waardenburg syndrome present cochlear hypoplasia [59].

Cochlear hypoplasia is also considered a characteristic feature in Warsaw syndrome along with severe pre- and postnatal growth retardation, microcephaly, intellectual disability, facial dysmorphia, and sensorineural hearing loss. Biallelic pathogenic variants in DDX11 on molecular genetic testing are present in these patients [60]. Although all patients reported in the literature seem to be affected by cochlear hypoplasia [61,62], further evaluations revealed that at least for two of them [63] should have been labeled as incomplete partitions type 2 [64]. Cochlear hypoplasia was also identified in patients with Pallister–Hall syndrome that presents the concomitant presence of large hamartoma-like lesions of the hippocampus [50].

Cochlear hypoplasia type 4 is a frequent feature in ⲁ-dystroglycan-related muscular disorders, a term that includes Walker–Warburg syndrome, muscle-eye-brain disease, and Fukuyama congenital muscular dystrophy. All of them present brain malformations and ocular abnormalities, in addition to muscular dystrophy. A recent study showed that all patients with Walker–Warburg syndrome presented cochlear hypoplasia type 4 as a characteristic feature [65].

Studies conducted on mice showed that mutations in CHD7, a gene mutated in human CHARGE syndrome, lead to cochlear hypoplasia [66], while humans with CHARGE syndrome frequently present different anterior [67] and posterior labyrinth anomalies [29]. In addition, the ablation of TBX2 from the otocyst leads to cochlear hypoplasia in mouse models [68].

Additional experimental studies highlighted that HOXA1/HOXB1 double mutants have uniform hypomorphic development of the inner ear [69].

## 8. Posterior Labyrinth Anomalies

The abnormalities of the posterior labyrinth are a wide spectrum of anomalies not fully characterized yet. The anterior labyrinth has always captured most of the attention because of the impact of a congenital hearing loss in the neuro-psycho-social evolution of human beings. On the other hand, motor and balance development seems to be less affected by congenital anomalies of the posterior part of the labyrinth because of the possible compensatory role of the visual and neurological systems. So, the role of these anomalies is still under evaluation.

Dysplasia of the semicircular canals, isolated or combined with cochlear anomalies, is described in several syndromes such as CHARGE syndrome, trisomy 13, and trisomy 18 [70]. In CHARGE syndrome it is typical to detect abnormalities in all the semicircular canals [71]. On the other hand, a partial involvement can be observed in trisomy 13, in which lateral semicircular canal defects are most common and are present with or without superior or posterior semicircular canal abnormalities, while in trisomy 18, the defects frequently affect the lateral and superior semicircular canals [72]. Isolated agenesis of the posterior semicircular canals, without involvement of the lateral semicircular canals, is extremely rare but has been detected in Waardenburg syndrome type II patients [73].

Bilateral anomalies of the posterior semicircular canals, with frequent concomitant involvement of the superior semicircular canals but normal lateral semicircular canals, were also described by Okuno in 1990 in the temporal bones of four individuals with Alagille syndrome [74]. This condition is characterized by chronic cholestasis, characteristic facial appearance, cardiovascular abnormalities, vertebral arch defect, growth retardation, mental retardation, and hypogonadism [75]. This syndrome is caused by mutations in the JAG1 gene [76]. According to Koch et al. the observation of a similar condition in Waardenburg syndrome type II suggests that this gene may have a crucial role for the development of the posterior semicircular canal, but not for the lateral semicircular canal that has a later development [70].

In some patients with Warsaw breakage syndrome, semicircular canal anomalies have been detected [62].

## 9. Conclusions

The identification of an inner ear malformation should suggest a targeted genetic investigation. Moreover, in these cases, the clinical evaluation should be extended beyond the audiological evaluation to exclude the possible presence of a syndromic condition. Conversely, in some cases the identification of specific gene mutations should lead to a neuroradiological evaluation to investigate the possible presence of anomalies of the labyrinth.

Clinicians involved in audiology research should be aware of the importance of neuroradiological evaluation and genetic consultation and testing to better assess the overall clinical status of hearing-impaired patients.

## Figures and Tables

**Table 1:** Sennaroglu’s classifications of inner ear malformations.

Sennaroglu (2002)	Sennaroglu (2017)	Subgroups (2017)	Description
Michel deformity	Complete labyrinthine aplasia	With hypoplastic or aplastic petrous bone	Absence of the whole cochleovestibular structure
		With otic capsule	
		Without otic capsule	
	Rudimentary otocyst	No subgroups	Incomplete and small representation of the otocyst without an internal auditory canal
Cochlear aplasia	Cochlear aplasia	With normal labyrinth	Absence of the cochlea
		With a dilated vestibule (CAVD)	Absence of the cochlea, bust the vestibule is enlarged
Common cavity deformity	Common cavity	No subgroups	Single, round chamber, representing cochlea and vestibule without any differentiation between them
Incomplete partition type 1	Incomplete partitions of the cochlea (differentiation of cochlea and vestibule, normal external dimensions)	Incomplete partition type 1	The entire modiolus and interscalar septa are absent, resulting in a cystic appearance of the cochlea
Incomplete partition type 2		Incomplete partition type 2	Absence of the apical part of the modiolus and corresponding interscalar septa, fusion of middle and apical turns
		Incomplete partition type 3	Absent modiolus but complete interscalar septa, enlarged internal acoustic canal
Cochlear hypoplasia (corresponding to CH-I)	Cochlear hypoplasia (external cochlear dimensions are smaller than normal)	Type1	Cochlea is like a small bud
		Type 2	Defective modiolus and interscalar septa, normal external outline
		Type 3	Short modiolus and less than 2 turns, reduced length of the interscalar septa
		Type 4	Normal basal turn, middle and apical turns are severely hypoplastic and located anteriorly and medially rather than in the center

**Table 2:** Summary of syndromes and genes related to each inner ear malformations.

Type of Malformation	Possible Related Syndrome	Genes Involved
Complete labyrinthine aplasia	Wildervanck syndrome	-
	LAMM syndrome	FGF3
	HOXA1 mutation syndrome	HOXA1
Rudimentary otocyst	-	-
Common cavity	Bosley–Salih–Alorainy syndrome	HOXA1
	22q11 deletion syndrome	-
	-	ROR1
Cochlear aplasia	-	PAX2 (animal studies)
Incomplete partition type 1	-	-
Incomplete partition type 2	Pendred syndrome	SLC26A4
	-	FOXI1
	-	KCNJ10
Incomplete partition type 3	-	POUF3F4
Cochlear hypoplasia	Branchio-oto-renal syndrome	-
	Oculo-auriculo-vertebral spectrum disorder	-
	Warsaw breakage syndrome	DDX11
	Pallister–Hall syndrome	-
	-	SIX1
	-	CHD7 (animal studies)
	-	TBX2 (animal studies)
	-	HOXA1/HOXB1 (animal studies)
Cochlear hypoplasia Type 1	-	-
Cochlear hypoplasia Type 2	-	-
Cochlear hypoplasia Type 3	Waardenburg syndrome	SOX10
Cochlear hypoplasia Type 4	ⲁ-Dystroglycan-related muscular disorders (Walker–Warburg syndrome)	-
Posterior labyrinth anomalies	CHARGE syndrome	-
	Trisomy 13	-
	Trisomy 18	-
	Waardenburg syndrome type II	-
	Alagille syndrome	JAG1
	Warsaw breakage syndrome	-
